# Knowledge and associated factors of healthcare professionals in detecting patient-ventilator asynchrony using waveform analysis at intensive care units of the federal public hospitals in Addis Ababa, Ethiopia, 2023

**DOI:** 10.1186/s12912-024-02068-8

**Published:** 2024-06-11

**Authors:** Habtamu Zelalem, Migbar Mekonnen Sibhat, Abate Yeshidinber, Habtamu Kehali

**Affiliations:** 1 Saint Paul’s Hospital Millennium Medical College, Addis Ababa, Ethiopia; 2 College of Health Sciences and Medicine, Dilla University, Dilla, Ethiopia

**Keywords:** Intensive care unit, Patient-ventilator asynchrony, Mechanical ventilation, Knowledge, Waveform analysis

## Abstract

**Background:**

The interaction between the patient and the ventilator is often disturbed, resulting in patient-ventilator asynchrony (PVA). Asynchrony can lead to respiratory failure, increased artificial ventilation time, prolonged hospitalization, and escalated healthcare costs. Professionals’ knowledge regarding waveform analysis has significant implications for improving patient outcomes and minimizing ventilation-related adverse events. Studies investigating the knowledge of healthcare professionals on patient-ventilator asynchrony and its associated factors in the Ethiopian context are limited. Therefore, this study aimed to assess the knowledge of healthcare professionals about using waveform analysis to detect asynchrony.

**Methods:**

A multicenter cross-sectional study was conducted on 237 healthcare professionals (HCPs) working in the intensive care units (ICUs) of federal public hospitals in Addis Ababa, Ethiopia, from December 2022 to May 2023. The data were collected using a structured and pretested interviewer-administered questionnaire. Then, the collected data were cleaned, coded, and entered into Epi data V-4.2.2 and exported to SPSS V-27 for analysis. After description, associations were analyzed using binary logistic regression. Variables with a *P*-value of < 0.25 in the bivariable analysis were transferred to the multivariable analysis. Statistical significance was declared using 95% confidence intervals, and the strengths of associations were reported using adjusted odds ratios (AORs).

**Results:**

A total of 237 HCPs participated in the study with a response rate of 100%. Half (49.8%) of the participants were females. The mean age of the participants was 29 years (SD = 3.57). Overall, 10.5% (95% CI: 6.9–15.2) of the participants had good knowledge of detecting PVA using waveform analysis. In the logistic regression, the number of MV-specific trainings and the training site had a statistically significant association with knowledge of HCPs. HCPs who attended more frequent MV training were more likely to have good knowledge than their counterparts [AOR = 6.88 (95% CI: 2.61–15.45)]. Additionally, the odds of good knowledge among professionals who attended offsite training were 2.6 times higher than those among professionals trained onsite [AOR = 2.63 (95% CI: 1.36–7.98)].

**Conclusion:**

The knowledge of ICU healthcare professionals about the identification of PVA using waveform analysis is low. In addition, the study also showed that attending offsite MV training and repeated MV training sessions were independently associated with good knowledge. Consequently, the study findings magnify the relevance of providing frequent and specific training sessions focused on waveform analysis to boost the knowledge of HCPs.

**Supplementary Information:**

The online version contains supplementary material available at 10.1186/s12912-024-02068-8.

## Introduction

Annually, approximately 20 million critically ill patients require admission to the intensive care unit and mechanical ventilation [1]. The global number of ventilator-assisted individuals (VAIs) ranges from 6.6 to 23 per 100,000. Maintaining a harmonious interaction between the patient and the ventilator requires adjusting the ventilator settings. Patient-ventilator asynchrony (PVA) is a frequent encounter during mechanical ventilation, affecting up to 80% of mechanically ventilated critical patients [2, 3]. Severe PVA (i.e., asynchrony index > 10%) is detected in approximately 25% of critically ill patients requiring artificial ventilation with mechanical ventilators [4, 5].

Despite the lack of well documented data regarding the variation in the detection of PVA between developed and resource limited settings, evidence showed that the effectiveness of mechanical ventilation and PVA detection is influenced by many factors including the income level and well-equipped settings [6]. Averagely, 21% of healthcare professionals working in the intensive care units (ICU) of developed settings correctly detected PVA whereas the rate of PVA detection in low- and middle-income countries (LMICs) was less than 15% [6–8].

PVA has been associated with increased morbidity and mortality [9]. PVA can lead to several negative health consequences, including discomfort, pain, decreased quantity and quality of sleep, and prolonged ICU stay. It may also result in ineffective ventilation, leading to increased respiratory effort, further respiratory distress (dyspnea), worsening of pulmonary gas exchange, and respiratory failure up to death. Moreover, PVA can result in an increased workload on clinicians and an escalation of healthcare costs to the patient and the healthcare system due to the increased sedation requirement and prolonged use of mechanical ventilators [3, 10–12].

PVA can be assessed and monitored either invasively or noninvasively. Waveform analysis, a noninvasive, inexpensive, and reliable method of detecting PVA, involves examining the shape of waveforms during different phases of the respiratory cycle (inspiration, transition from inspiration to expiration, and expiration) [13]. The identification and management of patient-ventilator asynchrony requires sophisticated respiratory physiology expertise. It is critical in caring for mechanically ventilated patients [14, 15]. However, studies conducted abroad have shown that healthcare providers working in intensive care units have a significant knowledge gap in recognizing PVA using waveform graphics monitoring, leading to suboptimal patient outcomes and increased healthcare costs [6, 8, 16, 17].

Various factors have been hypothesized to influence HCPs’ knowledge of waveform monitoring to recognize PVA, such as attending MV training, specific education and training on PVA and waveform graphics, completing PVA-related courses with more than 100 h load, the number of recognized PVAs, experience, and the number of ICU beds [6, 7, 18, 19].

Despite the relevance of skills and knowledge in waveform analysis to identify PVA, studies examining the knowledge of ICU healthcare professionals on PVA and its associated factors in the Ethiopian context are limited. The existing studies in Ethiopia investigated the knowledge and practice of HCPs regarding mechanical ventilation management and its associated factors. However, none of these studies addressed the concept of PVA and waveform analysis [20, 21]. Therefore, this study aims to assess the knowledge of HCPs toward identifying PVA using waveform graphics monitoring and its associated factors among professionals working in the ICU settings of Addis Ababa federal public hospitals, Ethiopia. Therefore, healthcare professionals need to have a good ability to detect and manage PVA.

## Methods

### Study design, area, and study period

A multicenter cross-sectional study was conducted in Addis Ababa federal public hospitals. Addis Ababa is the capital city of Ethiopia. According to the 2023 United Nations (UN) world population prospects, the city has an estimated population of 5,461,000 in 2022 (UN, 2023). There are six federal public hospitals in the city. These include St. Paul’s, Saint Peter, AaBET, ALERT, Black Lion, and Eka Kotebe hospitals. Approximately 271 professionals were assigned and working in the ICUs of the five federal public hospitals, which contain more than 75 well-equipped ICU beds, excluding Eka Kotebe. Eka Kotebe Hospital was not considered since the hospital was shifted recently from COVID-19 care provision. Hence, regular ICU service provision was not yet fully reinstalled during the study period.

As per the hospitals’ human resources data, St. Paul’s Millennium Medical College (SPHMMC) contains 21 ICU beds (14 adult ICUs and seven pediatrics ICUs). The Black Lion Specialized Hospital (BLH) has 18 ICU beds (12 adult ICUs and six pediatric ICUs). The Addis Ababa Burn, Emergency, and Trauma Centre (AaBET) has two ICUs with an 11-bed capacity. The ALERT Hospital Trauma Center has ICU departments with 12 well-equipped beds and trained nurses. St. Peter’s Specialized Hospital has ICU departments with 13 functional beds. The study was conducted from December 2022 to July 2023.

### Population of the study and eligibility criteria

The source population comprised all health care professionals (HCPs) working in the ICUs of federal public hospitals in Addis Ababa, Ethiopia. The study population was all healthcare professionals working in the ICUs of selected federal public hospitals in Addis Ababa during the data collection period. All health care professionals working in the ICU. The study excludes HCPs in the ICUs who were not directly involved in the mechanical ventilator manipulation, such as clinical pharmacists, dietitians, and physiotherapists. Healthcare professionals who were on medical or maternity leave and who had ICU experience of less than three months were excluded from the study.

### Sample size determination and sampling procedures

The sample size for this study was determined using a single population proportion formula by considering a 95% confidence interval, 5% degree of freedom, and proportion of knowledge of HCPs toward PVA detection, *p* = 12% from the study conducted in Alexandria, Egypt [8]. This brings 163 samples. After adding a 10% nonresponse rate, the required sample size for this study was 180. However, according to the data obtained from the respective hospitals’ human resource offices, there were only 271 healthcare professionals working in the ICUs of the selected hospitals. Of those, 34 healthcare professionals did not fulfill the inclusion criteria due to either no willingness to participate or absence during the data collection period. Accordingly, 237 healthcare professionals working in the ICUs of the selected hospitals fulfilled the inclusion criteria and incorporated to the study. Hence, we decide to consider the whole population since the study population was feasible (time and cost-effective) as well as easily manageable (not much higher than the calculated sample size). Besides, census can provide more accurate and precise data compared to sampling data. Therefore, it might ensure we get more representative and reliable data.

**Fig. 1:**
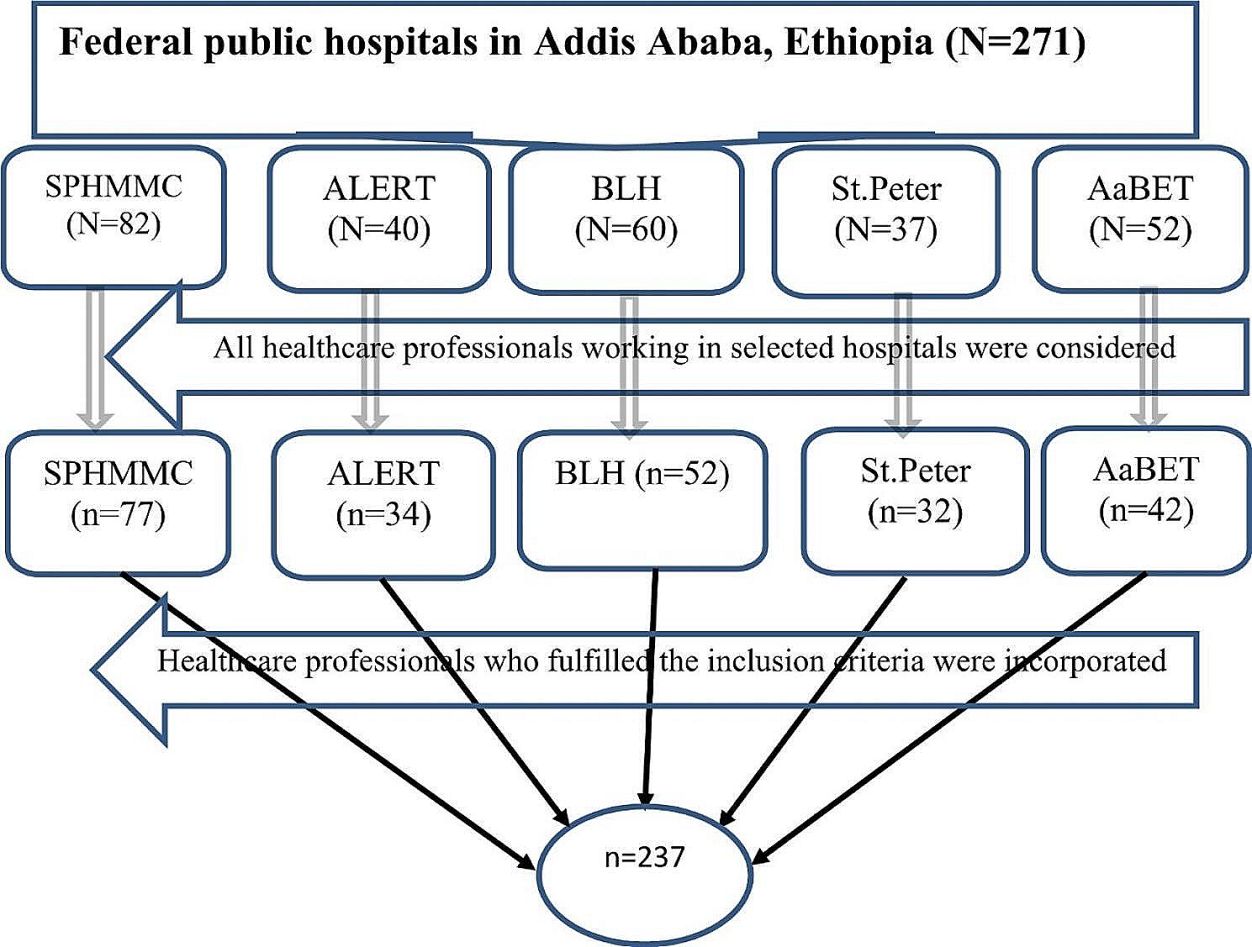
Schematic presentation of the sampling procedure of the study participants to assess the knowledge of HCPs toward PVA detection using waveform analysis

Afterward, the remaining 237 healthcare professionals were incorporated for the interview. Before the data collection, the sample size (*n* = 237) was proportionally allocated to each selected hospital. Consequently, 77 HCPs were selected from SPHMMC, 32 from Saint Peter’s Hospital, 34 from ALERT Hospital, 42 from AaBET Hospital, and 52 from Black Lion Hospital. The details of the sampling procedure (participant selection) are provided elsewhere (Fig. 1).

### Data collection tool and procedures

The data were collected using a structured interviewer-administered questionnaire. The tool was designed after reviewing different literature, ICU guidelines, and ICU textbooks [3, 7, 9, 19, 22]. The tool comprised sociodemographic characteristics, institutional, professional and training-related variables, and a knowledge assessment tool. Knowledge of HCPs on PVA was assessed using standardized pictures. The pictures included common types of patient-ventilator asynchronies (PVAs), such as missed triggering, delayed cycling, flow asynchrony, double triggering, auto triggering, and a demonstration of normal ventilator waveform. The HCPs were asked to identify the presence or absence of PVA using seven-item knowledge assessment ‘yes’ or ‘no’ questions and, if present, to specify the type of PVA by choosing the best response from a multiple-choice question in the assessment sheet (supplementary file). The data collection was undertaken from March to May 2023.

### Quality control

The tool was validated by a group of experts (a combination of intensivists, anesthesiologists, respiratory therapists, and research experts participated), and approved for use such that it could attain the desired objective. The item consistency (reliability) for knowledge assessment questions was assessed using Cronbach’s Alpha, and it was acceptable (α = 77.3%). The questionnaire was pretested on 5% of randomly selected samples at Eka Kotebe Hospital for clarity and consistency. Five nurses (one nurse for each hospital) were recruited to collect the data, and two MSc fellows monitored and supervised the data collection process. Half-day training was given for data collectors before proceeding to data collection. The principal investigator audited the collected data daily and ensured the completeness and validity of the responses.

### Operational definitions

#### Training

HCPs are considered trained if they have completed at least one training session focusing (entirely) on mechanical ventilation. **Onsite** training refers to training given within the working institution for the staff, whereas **offsite** training refers to training provided in the training centers other than the institution where the trainers were working. Time lapsed after the last training ever attended in this study was classified as < one year and ≥ one year [6, 9]. **Knowledge** of healthcare professionals was assessed using a seven-item knowledge assessment tool. The total knowledge score of HCPs was computed using a spreadsheet by summing up the individual responses. The modified Bloom’s cutoff point, a tool used to categorize healthcare providers’ knowledge levels, was applied based on their performance on a knowledge assessment test. In this tool, healthcare providers who scored above or equal to 75% of the total expected knowledge score were categorized as having good knowledge, whereas those who scored less than 75% of the total expected knowledge score were declared as having poor knowledge [19, 23].

### Data analysis

Data were checked, coded, and entered into Epi data V-4.2.2 and exported to SPSS V-27 for analysis. Before analysis, the nature of the data distribution, outliers, and missing values were determined. Then, continuous variables are presented as the mean with standard deviation (SD) or median with interquartile range (IQR). On the other hand, categorical variables were described using frequency tables, texts, and graphs. After description, bivariable and multivariable analyses were run using a binary logistic regression model to assess the possible association between the dependent and independent variables. Bivariable analysis was conducted to determine the association of each independent variable with the dependent variable to recruit the candidate variables for the final model. Variables with a *P*-value of 0.25 in the bivariable analysis were fitted to multivariable analysis for confounder adjustment. Multi-collinearity was checked using variance inflation factor (VIF) with a minimum of 1.207 and a maximum of 2.032 (mean VIF = 1.483), which indicates as no evidence of collinearity. Model fitness was checked using the Hosmer and Lemshew test, and the output was *p* = 0.162. Finally, statistical significance was declared using a 95% confidence interval, and adjusted odds ratios were used to report the strength of associations between the dependent and independent variables.

### Ethical consideration

Written ethical clearance was obtained from St. Paul’s Hospital Millennium Medical College institutional review board (IRB). A letter of cooperation for data collection was written for each selected hospital, and permission for data collection was received from the selected hospitals to conduct the research. Before data collection, the objective of the study was clearly explained to the study participants. Furthermore, the participants were informed that no individual benefits or perceived risks were involved upon participation. After agreeing to participate, written informed consent was obtained from each participant. The study was conducted as per the Declaration of Helsinki. The study participants were informed that they were free to ask any question about the data collection or could depart anytime from the study during the data collection process if they deemed to do so, and reasons for withdrawal were not asked. All the collected data will be kept confidential, and no one except the investigators will have access to the collected information. The autonomy and privacy of the participants were assured by aggregate reporting and removing personal identifiers.

## Results

Of the 271 healthcare professionals (HCPs) currently employed in the intensive care units (ICUs) of selected federal public hospitals, 237 met the inclusion criteria and participated in this study. The remaining 34 HCPs were either unavailable during the data collection period or declined to participate and, thus, were excluded.

### Sociodemographic characteristics of HCPs

Half, (49.8%), of the study participants were female. The mean age of the participants was 29 years (SD = 3.57). The minimum age of the participants’ was 23 years, and the maximum was 42 years; only 68 (26%) of them were aged above 30 years. 17% (40) of the participants had a second degree or above (Table 1).

**Table 1:** Sociodemographic and institution-related characteristics of HCPs working in the ICU settings of federal public hospitals in Addis Ababa, Ethiopia, 2023 (*n* = 237)

Independent variables	Category	Knowledge of HCPs		Total (%)
		Good (%)	Poor (%)	
Age	< 30 years	11 (6.5)	158 (93.5)	169 (100)
	>=30 years	14 (20.6)	54 (79.4)	68 (100)
Sex	Male	16 (13.4)	103 (86.6)	119 (100)
	Female	9 (7.6)	109 (92.4)	118 (100)
Educational level	1st degree	12 (6.1)	185 (93.9)	197 (100)
	2nd degree	13 (32.5)	27 (67.5)	40 (100)
Institution	SPHMMC	10 (13.0)	67 (87.0)	77 (100)
	St. Peter	0 (0.0)	32 (100)	32 (100)
	BLH	5 (9.6)	47 (90.4)	52 (100)
	ALERT	0 (0.0)	34 (100)	34 (100)
	AaBET	10 (23.8)	32 (76.2)	42 (100)
Type of ICU	Adult ICU	21 (10.7)	176 (89.3)	197 (100)
	Pediatrics ICU	4 (10.5)	36 (89.5)	40 (100)

**Abbreviations**: HCPs, healthcare professionals; RT, SPHMMC, Saint Paul’s Hospital Millennium Medical College; BLH, Black Lion Hospital; ALERT, All African Leprosy and Tuberculosis Rehabilitation and Training Center; AaBET, Addis Ababa Burn Emergency and Trauma Hospital; ICU, intensive care unit

### Profession and training-related characteristics of HCPs

Professionals who work in the pediatric ICU (PICU) and adult ICU settings of each selected hospital were invited to participate in this study. Accordingly, forty (16.9%) participants were working in the pediatric ICU (Table 1). Regarding the participants’ profession, almost one-fifth (19.4%) were physicians, and 146 were nurse professionals. On the other hand, the median ICU working experience was two years (interquartile range, IQR = 1), and the maximum recorded ICU experience was eight years. Only 10% of the respondents served for five years and above in the ICU settings, and 75% had experience not exceeding three years (Table 2).

**Table 2:** Distribution of profession and training-related characteristics of HCPs working in the ICU settings of federal public hospitals in Addis Ababa, Ethiopia, 2023 (*n* = 237)

Parameters	Category	Knowledge of HCPs		Total (%)
		Good (%)	Poor (%)	
Profession of the HCPs	Physician	13 (28.3)	33 (71.7)	46 (100)
	Nurse	9 (4.8)	178 (95.2)	187 (100)
Specialty of the HCPs	RT	3 (75.0)	1 (25.0)	4 (100)
	Intensivist	2 (66.7)	1 (33.3)	3 (100)
	Anesthesiologist	1 (14.3)	6 (85.7)	7 (100)
	EMCC specialist	7 (38.9)	11 (61.1)	18 (100)
	Pediatrician	3 (37.5)	5 (62.5)	8 (100)
	GP	0 (0.0)	10 (100)	10 (100)
	ECCN	3 (7.3)	38 (92.7)	41 (100)
	Generic nurse	6 (4.1)	140 (95.9)	146 100)
	RT	3 (75.0)	1 (25.0)	4 (100)
ICU experience	≤ 3years	20 (11.2)	159 (88.8)	179 (100)
	> 3years	5 (8.6)	53 (91.4)	58 (100)
Specific training on MV	Yes	24 (13.1)	159 (86.9)	183 (100)
	No	1 (1.9)	53 (98.1)	54 (100)
Site of MV training held	Offsite	15 (35.7)	27 (64.3)	42 (100)
	Onsite	9 (6.4)	132 (93.6)	141 (100)
Does previous MV training contain PVA and waveform analysis?	Yes	11 (35.5)	20 (64.5)	31 (100)
	No	13 (8.6)	139 (91.4)	152 (100)
Time lapsed after the last training ever attended	≤ 1 year	15 (16.1)	78 (83.9)	93 (100)
	>1 year	9 (10.0)	81 (90.0)	90 (100)

***Abbreviations***: *HCPs, healthcare professionals; RT, respiratory therapist; EMCC, emergency medicine and critical care specialist; GP, general practitioner; ECCN, emergency and critical care nurse; ICU, intensive care unit; MV, mechanical ventilator; PVA, patient-ventilator asynchrony*

**Fig. 2:**
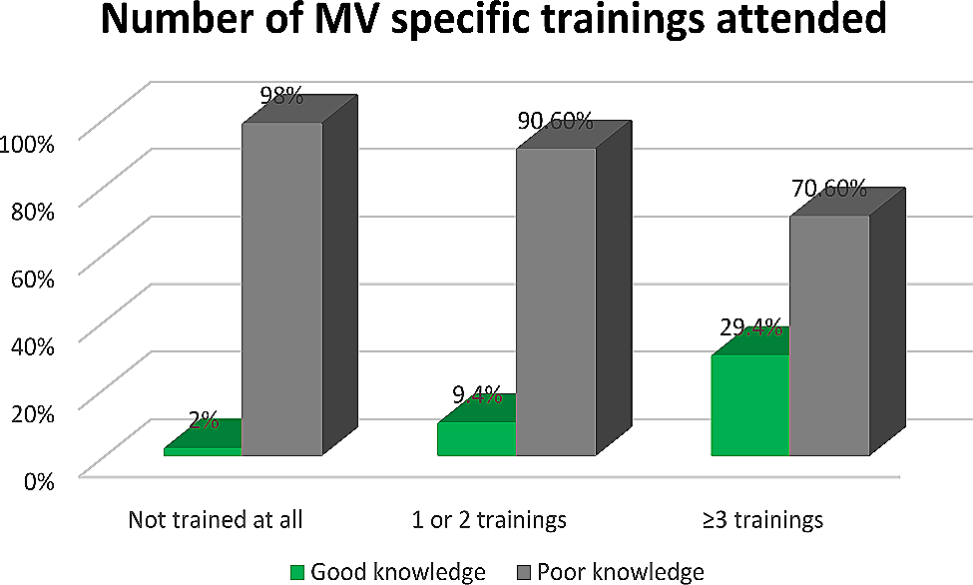
Number of MV-specific trainings attended by HCPs working in the ICUs of federal public hospitals in Addis Ababa, Ethiopia, 2023 (*n* = 237)

Approximately 29.4% of HCPs who received three or more MV trainings had good knowledge of PVA detection using waveform analysis, whereas only 2% of those who never trained for MV had good knowledge (Fig. 2).

### Distribution of MV-training-related characteristics among HCPs

The majority (77.2%) of the study participants received mechanical ventilator training at least once. However, less than 17% of those trained responded that the session included patient-ventilator asynchrony (PVA) and waveform analysis. Of the 183 HCPs who received MV management training, 14.3% (34/183) attended three or more training sessions, with a maximum of six sessions. Furthermore, more than 50% of the HCPs attended just one MV-related training session. Regarding the training site, just 23% of the participants trained fully off the job (offsite) (Table 2).

### Description of HCPs’ knowledge of PVA detection using waveform analysis

**Fig. 3:**
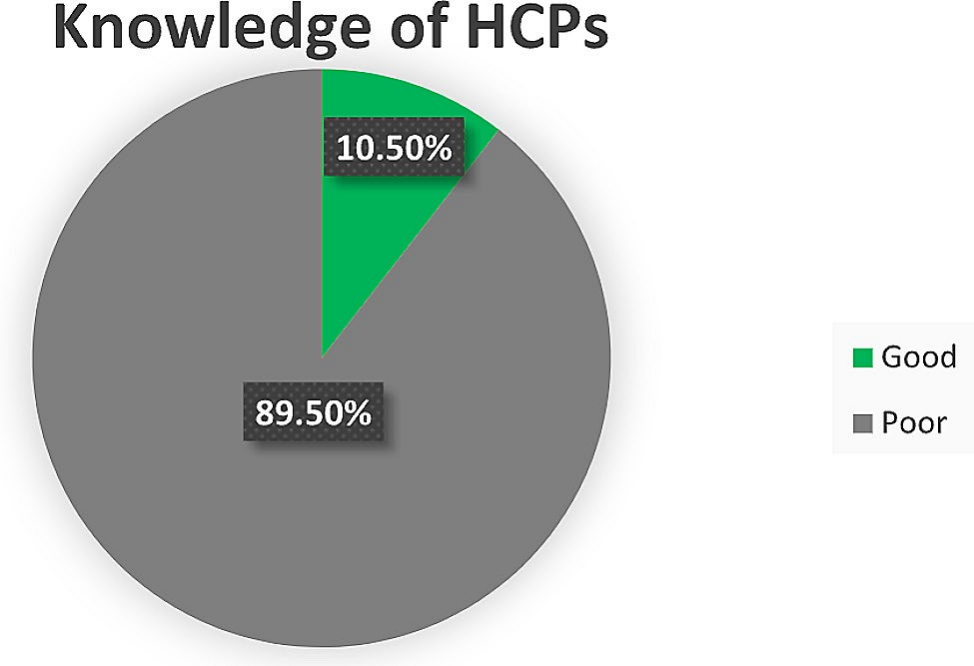
Knowledge of PVA identification using waveform analysis among HCPs working in the ICU settings of federal public hospitals in Addis Ababa, Ethiopia, 2023 (*n* = 237)

According to the study findings, 10.5% (95% CI: 6.9–15.2) of HCPs were found to have good knowledge, whereas 89.5% (95% CI: 84.8–93.1) had poor knowledge of detecting PVA using waveform analysis (Fig. 3).

The proportion of good knowledge among males was 13.4%, whereas 7.6% of female participants had good knowledge (*p* = 0.145). Besides, approximately 32.5% of participants with an educational level of 2nd degree or above had good knowledge. Conversely, only 6.1% of 1st degree holders had good knowledge (*p* < 0.001) (Table 1). Among HCPs who had good knowledge, 96% took specific MV training, whereas only 4% of HCPs had good knowledge without attending MV-specific training (*p* = 0.018). On the other hand, among those MV trained, the proportion of good knowledge was much higher (35.5%) among HCPs who took MV training inclusive of PVA and waveform analysis compared to their counterparts (8.5%, *p* < 0.001). HCPs who spent a longer time (more than one year) after the last training ever attended had a relatively lower level of knowledge (6.7%) compared to those who spent less time (16.1%, *p* = 0.219) (Table 2).

### Factors associated with the level of knowledge of HCPs toward PVA detection using waveform analysis

After bivariable analysis, variables such as age, sex, educational level, profession, ICU experience, number of MV trainings attended, site of training, PVA and waveform analysis containing MV training courses, and time elapsed after the last training session were fitted to multivariable analysis for confounder adjustment. Afterward, the number of MV trainings attended and offsite trainings attended was found to have a statistically significant association with the level of knowledge at the 95% confidence level (Table 3).

HCPs who attended more MV management training sessions were more likely to have good knowledge than their counterparts [AOR = 6.88 (95% CI: 2.61–15.45)]. This means that the odds of having good knowledge of PVA detection among HCPs who attended three or more training sessions was 6.9 times higher compared to those who attended less than two training sessions or not at all. Likewise, attending offsite MV training showed a statistically significant association with knowledge of HCPs [AOR = 2.63 (95% CI: 1.36–7.98)]. Professionals who attended MV training off the job (offsite trained) were 2.6 times more likely to have good knowledge compared to HCPs who attended training in their institution while on the job (onsite trained) (Table 3).

**Table 3:** Bivariable and multivariable logistic regression analysis output for the factors associated with knowledge of HCPs toward PVA detection using waveform analysis among HCPs working in the ICU settings of federal public hospitals in Addis Ababa, Ethiopia, 2023 (*n* = 237)

Independent variable	Category	Knowledge of HCPs		COR (95% CI)	AOR (95% CI)	*P*-value
		Good	Poor			
Sex	Male	16	103	1.88 (0.80–4.45)	4.40 (0.98–19.85)	0.054
	Female	9	109	Reference	Reference	
Age	< 30 years	11	158	Reference	Reference	
	≥ 30 years	14	54	3.72 (1.60–8.70)	2.42 (0.53–11.13)	0.255
Educational level	1st degree	12	185	Reference	Reference	
	2nd degree	12	27	7.42 (3.07–17.94)	0.62 (0.12–3.14)	0.560
ICU experience	≤ 3years	20	159	Reference	Reference	
	> 3years	5	53	0.75 (0.27–2.10)	0.21 (0.04–1.17)	0.075
Number of MV training attended	≤ 2sessions	15	188	Reference	Reference	
	≥ 3sessions	10	24	5.22 (2.11–12.92)	6.88 (2.61–15.45)	0.001*
Site where MV training held	Offsite	15	27	8.15 (3.23–20.53)	2.63 (1.36–7.98)	0.001*
	Onsite	9	132	Reference	Reference	
Time lapsed after the last training ever attended	≤ 1 year	15	78	Reference	Reference	
	> 1 year	9	81	0.58 (0.24–1.40)	1.72 (0.46–6.40)	0.417
MV training contains PVA and waveform analysis	Yes	11	20	5.88 (2.32–14.90)	0.51 (0.15–1.72)	0.278
	No	13	139	Reference	Reference	
Type of Profession	Physician	13	33	5.88 (2.47–13.99)	0.41 (0.11–1.51)	0.179
	Nurse	12	179	Reference	Reference	

*significant at the 95% confidence level

***Abbreviations***: *HCPs, healthcare professionals; COR, crude odds ratio; CI, confidence interval; AOR, adjusted odds ratio; ICU, intensive care unit; MV, mechanical ventilator; PVA, patient-ventilator asynchrony*

## Discussion

This study aimed to assess the knowledge of HCPs in using waveform analysis to detect PVA and to identify its associated factors. Accordingly, only 10.5% of HCPs had good knowledge. Furthermore, the number of MV trainings attended and the number of offsite trainings attended were independently associated with the level of knowledge.

The knowledge of HCPs in recognizing PVA using waveform graph analysis was too low. Only 10.5% (95% CI: 6.9–15.2) of the HCPs had good knowledge. This finding agrees with an earlier study conducted in Saudi Arabia, which showed that approximately 10.2% of healthcare workers can correctly detect PVA using waveform graphics [7]. Another study conducted in Alexandria, Egypt, by Abdelgawad et al. to assess critical care nurses’ knowledge of waveform monitoring to detect PVA similarly documented a low level of knowledge (12%) [8]. This low level of knowledge might be due to insufficient coverage of PVA and waveform graphics contents in the curriculum and mechanical ventilator and critical care training. To support this statement, less than 17% of the training sessions contained patient-ventilator asynchrony (PVA) and waveform analysis contents. This implies that most of the MV management training did not incorporate PVA detection and waveform analysis usage, as such professionals might be ineffective in detecting and implementing this relevant clinical aspect.

HCPs who attended more frequent MV management training sessions were more likely to have good knowledge than their counterparts [AOR = 6.88 (95% CI: 2.61–15.45)]. The findings also revealed that only 4% of HCPs had good knowledge without attending MV-specific training compared to 13.2% among those trained. Despite previous studies that did not document the number/frequency of training sessions, MV-specific training was identified to have a statistically significant association with the knowledge/ability of health professionals to monitor PVA using waveform graphs. Reports from Chile by Ramirez et al. [6], an international survey conducted on 20 countries by Ramirez II et al. [9], and from Spain by Chacon E et al. [24] supported this finding. In line with this, studies also noted a significant improvement in the knowledge of HCPs after PVA and graphics training. For instance, a quasi-experimental systematic review conducted in America by Lynch-Smith et al. reported a significant knowledge score improvement after PVA lectures were provided [25].

In addition, studies conducted in Italy [16], Chile [6], Spain [24], and Saudi Arabia [7] also supported this finding. In this study, we noted that HCPs who received PVA-containing MV training had much better knowledge (35.5%) than HCPs who received only MV training not containing PVA and graphics content (8.5%). The possible justification for this finding could be the evident effect of content-specific training on the knowledge of the topic of interest. Moreover, frequent training sessions contribute to improvement in the level of knowledge. This is because as HCPs are exposed repeatedly to PVA and waveform graphics monitoring, they will become familiar with this concept and could appreciate it simply.

On the other hand, professionals who attended MV training off the job (offsite trained) were 2.63 times more likely to have good knowledge compared to HCPs who trained in their institution while on the job (onsite trained). To the researcher’s knowledge, none of the former studies documented the association of training sites with knowledge of HCPs. The possible justification for this association might be the freedom of trainers. Off-the-job training might enable trainers to focus on the training session without being interrupted by their clinical duty. Besides, off-the-job training might provide adequate rest and boost mental readiness for the training session. Contrarily, onsite trainers might have a sense of exhaustion and interruption of training attendance in case of workloads and duty gaps, which limit their effectiveness and success in the new knowledge acquisition.

In this study, the proportion of good knowledge among HCPs with a 2nd degree and above (32.5%) was more than fivefold higher than that among 1st degree holders (6.1%). Nevertheless, no statistically significant association was depicted between sociodemographic, profession-related, and institutional characteristics and knowledge of HCPs. Several studies conducted abroad documented consistent findings [6, 7, 17]. A multicenter study conducted in China, Italy, and the Netherlands also conformed to our reports that no PVA detection differences were observed by expertise and interface [18].

Consequently, better knowledge of healthcare professionals (HCPs) to interpret waveform graphs as well as provision of frequent and comprehensive MV trainings containing waveform graph analysis packages would mean increased ability of PVA detection, which in turn contribute to increased successful MV support, reduced MV-associated complications, improved patient outcome, and decreased ICU mortality. Furthermore, it could also lead to reduced healthcare burden and cost to the patient as well as the healthcare system.

The study possesses several strengths that worth recognition. To the researchers’ knowledge, this study was the first of its kind to specifically assess HCP’s knowledge towards PVA detection in the ICUs and its associated factors in Ethiopia. Thus, it represents a significant contribution to the limited literature on PVA in the context of resource constraint countries where the impact of PVA on patient outcomes is presumed to be particularly pronounced due to limited access to resources and training. Nevertheless, certain limitations should be considered when interpreting the findings. First, the study was conducted in specialized and teaching hospitals and did not incorporate professionals working in primary and general hospitals. This might overestimate the level of knowledge. Furthermore, the study focused solely on the knowledge of healthcare professionals and did not evaluate their actual practices and outcomes in managing PVA in mechanically ventilated patients. Thus, further research may be required to investigate the knowledge, practices, and outcomes of managing PVA in different healthcare settings and regions.

## Conclusion

In summary, the study findings suggest that healthcare professionals’ knowledge regarding the identification of patient-ventilator asynchrony using waveform graphics analysis is too low. Besides, the study also showed that attending MV training outside of the working institution (offsite training) and attending repeated MV training sessions were independently associated with the level of knowledge. Consequently, the study findings magnify the relevance of providing frequent and focused training sessions to boost HCP knowledge of waveform analysis for PVA detection. Moreover, further research may be required to address the practice and application outcomes of HCPs’ knowledge.

### Electronic supplementary material

Below is the link to the electronic supplementary material.

Supplementary Material 1

## Data Availability

All relevant data are within the manuscript and its supporting information files.

## Abbreviations

PVA: Patient-ventilator asynchrony;
ICU: Intensive care unit;
MV: Mechanical ventilator/ventilation;
HCPs: Health care professionals;
SPHMMC: Saint Paul’s Hospital Millennium Medical College;
BLH: Black Lion Hospital;
AaBET: Addis Ababa burn emergency and trauma center;
ALERT: All African Leprosy and Tuberculosis rehabilitation and training center;
RT: Respiratory therapist;
EMCC: Emergency medicine and critical care specialist;
GP: General practitioner;
ECCN: Emergency and critical care nurse;
