# Supplementary material for: Knowledge and associated factors of healthcare professionals in detecting patient-ventilator asynchrony using waveform analysis at intensive care units of the federal public hospitals in Addis Ababa, Ethiopia, 2023
Source: BMC Nurs. 2024 Jun 11;23:398. doi: 10.1186/s12912-024-02068-8 (PMC11165806; doi:10.1186/s12912-024-02068-8)
Supplement: Supplementary file 1 — Supplementary Material 1 [file 12912_2024_2068_MOESM1_ESM.docx]

**Data collection tool**

**Part I: Sociodemographic Characteristics of study participants**

| 101. Age of the respondent | **____________** |
| --- | --- |
| 102. Gender of the respondent | 1. Male 2. Female |
| 103. Institution /hospital where the respondent is working | 1. SPHMMC  2. St Peter Hospital  3. Black Lion Hospital  4. ALERT Hospital  5. AaBET Hospital |
| 104. Specialty | 1. Intensivist 2. Anesthesiologist 3. EMCC Specialist 4. GP 5. Critical care Nurse 6. Generic Nurse 7. Respiratory Therapist 8. Other (Specify) |
| 105. Educational Level | PhD   1. MSc 2. BSc 3. Diploma |
| **Part II: Institution, profession and training-related characteristics of study participants** | |
| 201. Which ICU are you working now? | 1. Adult ICU 2. Pediatric ICU 3. Neonatal ICU |
| 202. Number of ICU beds in your hospital? | ___________ |
| 203. Patient to nurse ratio in your hospital? | __________ |
| 204. Work experience in ICU (doing invasive mechanical ventilation) in years? | __________ |
| 205. Did you get specific Training on Ventilator? | 1. yes 2. No |
| 206. If your answer for the above question is YES, how many MV related training sessions did you attend? | ____________ |
| 207. If your answer for the question 2.4 is YES Where is the Place training held? | 1. In school training  2. On job training |
| 208. When did you take the last training? | ____________ |
| 209. Does the pervious MV training contain patient ventilator synchrony and wave form analysis? | 1. Yes 2. No |

**Part III: Knowledge on Asynchrony**

1. Have you ever seen patient-ventilator mismatch (asynchrony)?
2. Yes
3. No
4. How do you define patient-ventilator asynchrony?
5. Optimal patient-ventilator interaction
6. Comfort with mechanical ventilation and to avoid poor outcomes
7. The mismatch between patient and ventilator.
8. I know nothing about asynchrony
9. Have you ever heard about types of asynchrony?
10. Yes
11. No
12. A patient is on mechanical ventilation, you observe the graphs below on the ventilator screen. Which type of asynchrony do you think is displayed in the figure below?


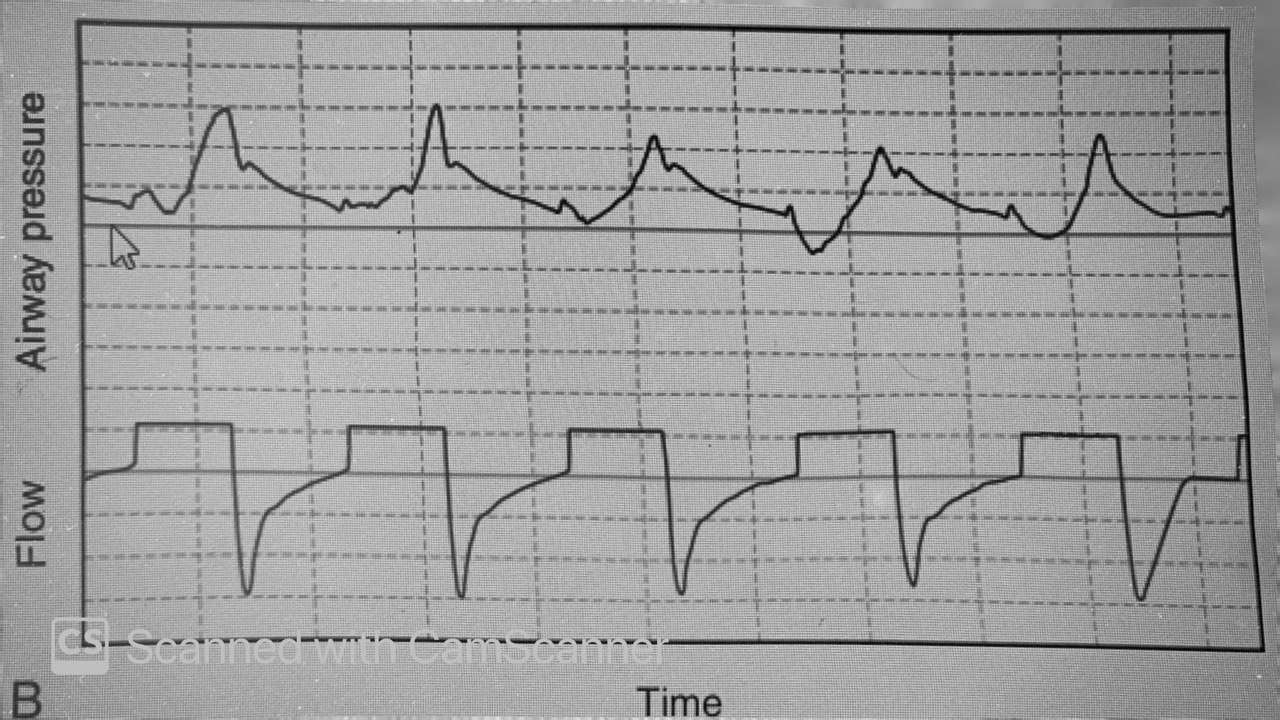


1. Delayed Cycling
2. Missed Triggering
3. Flow Asynchrony
4. Double Triggering
5. There is no Asynchrony
6. While the patient is on mechanical ventilation, you observe the graph below on the mechanical ventilator screen. Choose the correct types of asynchrony.


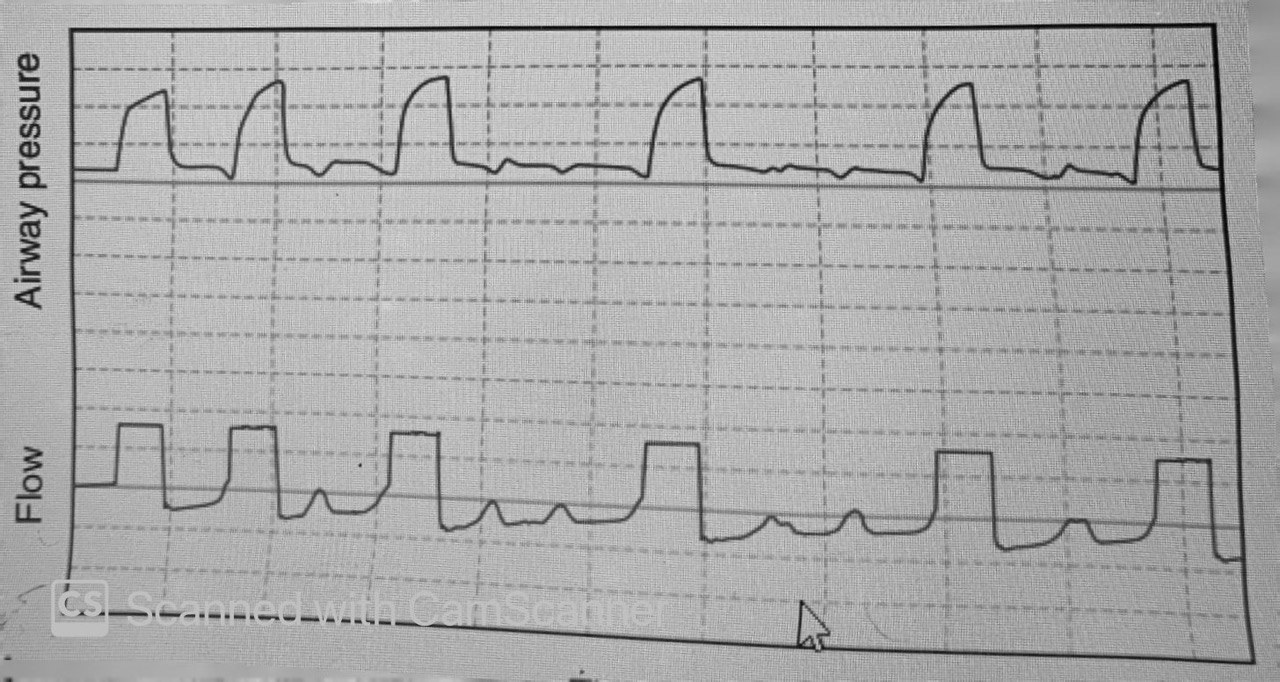


1. Auto Triggering
2. Delayed Cycling
3. Flow Asynchrony
4. Missed Triggering
5. There is no Asynchrony
6. While you are approaching a patient supported with mechanical ventilator for respiratory compromise, you observe the graph below on the ventilator screen. How could you document the output in the registry (Which type of asynchrony do you think is indicated in the figure below if asynchrony is present)?


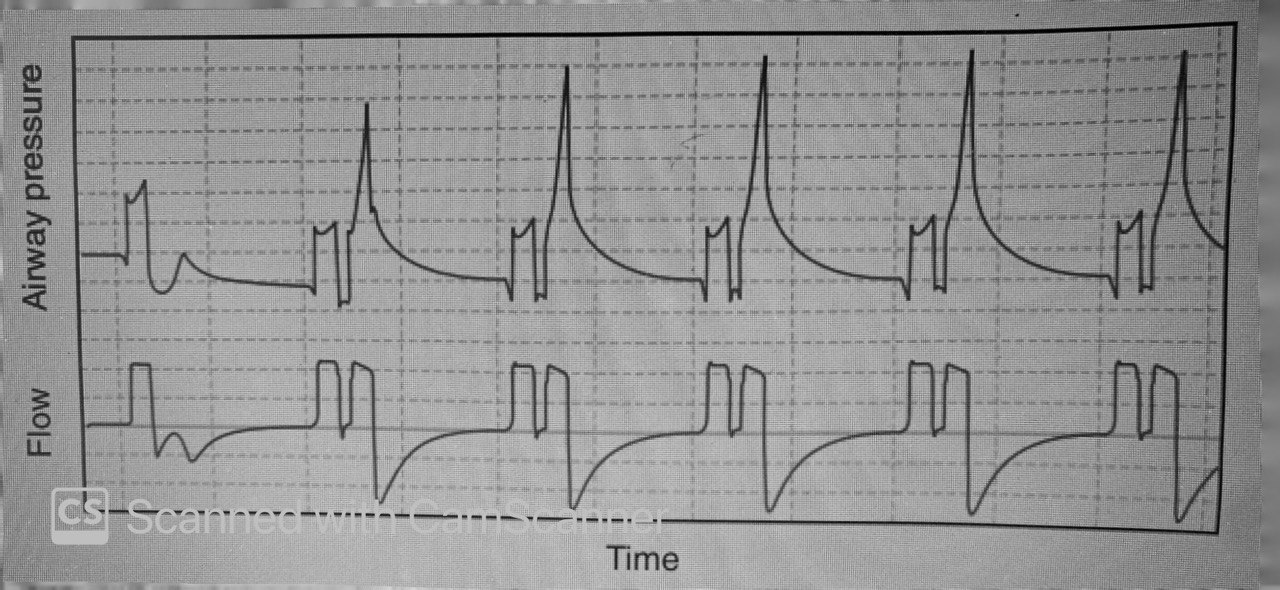


1. Double Triggering
2. Delayed Cycling
3. Flow Asynchrony
4. Auto Triggering
5. There is no Asynchrony
6. A patient is on mechanical ventilation, you observe the graphs below on the ventilator screen. Which type of asynchrony do you think is indicated in the figure below if asynchrony is present?


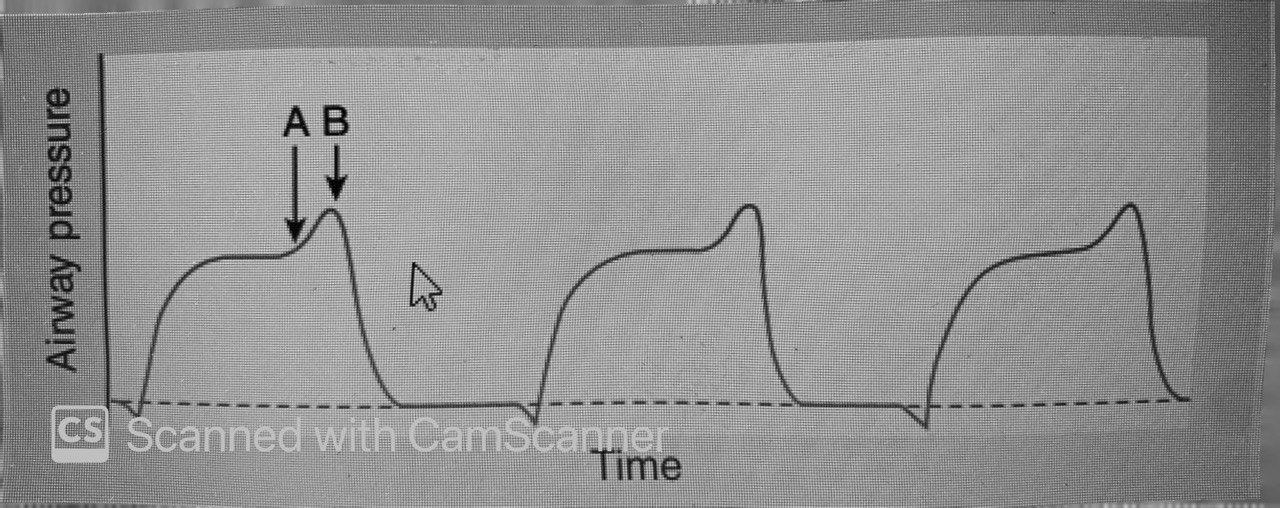


1. Double Triggering
2. Delayed Cycling
3. Flow Asynchrony
4. Auto Triggering
5. There is no Asynchrony
6. A patient is on mechanical ventilation, you observe the graphs below on the ventilator screen. Which type of asynchrony do you think is indicated in the figure below if asynchrony exist?


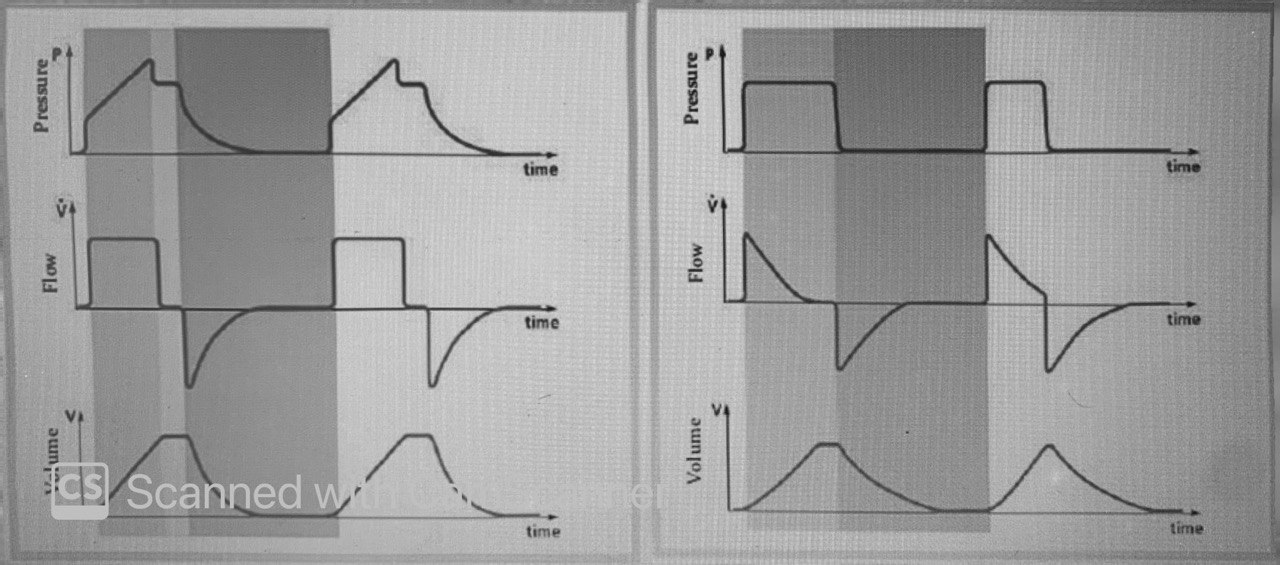


1. Double Triggering
2. Auto Triggering
3. Flow Asynchrony
4. Delay Cycling
5. There is no Asynchrony
6. A patient is on mechanical ventilation, you observe the graphs below on the ventilator screen. Choose the correct types of asynchrony.


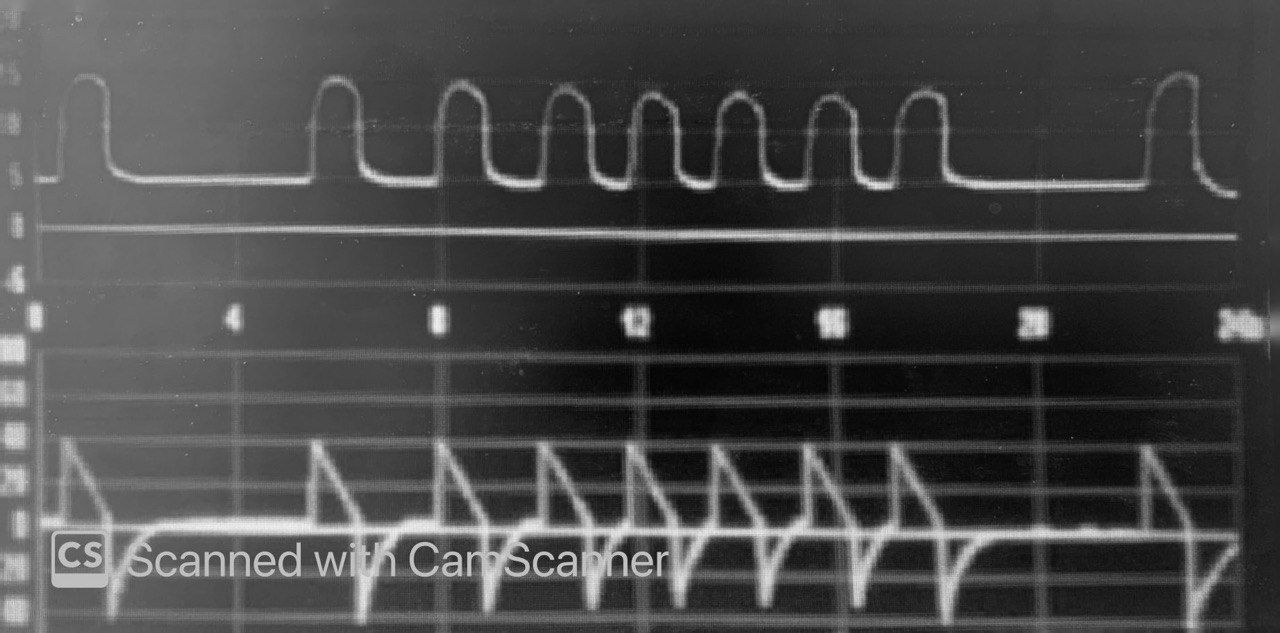


1. Auto Triggering
2. Delayed Cycling
3. Flow Asynchrony
4. Missed Triggering
5. There is no Asynchrony
